# Meiosis in the scorpion *Tityus silvestris*: new insights into achiasmatic chromosomes

**DOI:** 10.1242/bio.040352

**Published:** 2019-05-15

**Authors:** Bruno Rafael Ribeiro de Almeida, Renata Coelho Rodrigues Noronha, Marlyson Jeremias Rodrigues da Costa, Cleusa Yoshiko Nagamachi, Julio Cesar Pieczarka

**Affiliations:** Laboratório de Citogenética, Centro de Estudos Avançados em Biodiversidade, Instituto de Ciências Biológicas, Universidade Federal do Pará, Avenida Augusto Corrêa, s/n, 66075-900, Guamá, Belém, Pará, Brazil

**Keywords:** Holocentric chromosome, Achiasmatic meiosis, Scorpiones, Epigenetics, Synaptonemal complex

## Abstract

Achiasmatic male meiosis in scorpions is characterized by a high frequency of gaps, asynaptic regions and multivalent associations. Here, we performed an immunocytogenetic analysis to investigate recombination, and synapsis and chromatin-remodeling events during meiosis of the scorpion *Tityus silvestris*. Our results demonstrate that the synaptonemal complex (SC) begins its organization in the zygotene stage and persists until metaphase I. The advancement of the synaptic process is related to the epigenetic modification histone H3 lysine 27 trimethylation (H3K27m3). The distribution and dynamics patterns of variant γH2AX and recombinase Rad51 during achiasmatic meiosis suggests formation and repair of DNA double-strand breaks (DSBs) during early stages of prophase I. The epigenetic modifications, histone H3 lysine 4 trimethylation (H3K4m3) and histone H3 lysine 9 acetylation (H3K9ac), showed a dispersed distribution along the bivalents, suggesting that transcriptional activity is maintained constitutively during prophase I. However, H3K9ac modifications are absent in constitutive heterochromatin carrying the 45S rDNA in pachytene and post-pachytene stages. Collectively, our data demonstrate that *T. silvestris* exhibits adaptations to the achiasmatic mode, and suggest that epigenetic modifications may act in the regulation of these mechanisms to favor the normal continuation of meiosis in this scorpion.

## Abstract (precis)

**Summary:** The scorpion *Tityus silvestris*, despite not presenting chiasma, performs initial stages of the recombination process and maintains the homologues synapse and different epigenetic modifications until late stages of meiosis I.

## INTRODUCTION

*Tityus* is the largest genus of the Buthidae family in the Neotropical region, comprising about 200 described species, some of which are of great medical importance (Kovařík et al., 2013; Lourenço, 2002, 2015). In recent years, these scorpions have been the subject of several meiotic studies, reflecting the fact that they exhibit extensive multivalent associations during metaphase I. These meiotic chains may involve all chromosomes of the karyotype and are the result of the occurrence of structural rearrangements of the fusion/fission type or reciprocal translocations in the heterozygous state (Piza, 1950a,b; Cunha and Pavan, 1954; Adilardi et al., 2016; Ojanguren-Affilastro et al., 2017). The presence of holocentric chromosomes favors fixation of these rearrangements in several natural populations (Mattos et al., 2013). Direct consequences of such changes are detected during prophase I events, as evidenced by synaptic anomalies, such as gaps, asynapsed regions, loop-like structures and a high frequency of heterosynapsis, in several members of this genus (Schneider et al., 2009b).

Another interesting aspect of *Tityus* meiosis is the absence of chiasma in males (Schneider et al., 2009a). Achiasmatic meiosis is not unique to this genus, occurring in all Scorpiones families, as well as in other organisms, such as plants, flatworms and insects (Schneider et al., 2009a; Loidl, 2016). Chiasma formation is related to the occurrence of DNA double-strand breaks (DSBs), created by the enzyme Spo11 (Moens et al., 2007). The recombinases, Rad51 (radiation sensitive 51) and DMC1 (disrupted meiotic cDNA1), perform reciprocal repair by subsequently associating with regions of DSBs and connecting them to the homologous DNA strand, forming a heteroduplex (Gray and Cohen, 2016). The chiasma is the cytological visualization of this process during the diplotene stage, after dissociation of the synaptonemal complex (SC). To date, no detailed studies of meiotic recombination in scorpions have been reported, with the exception of an ultrastructural analysis of the SC of some Buthidae species that showed no recombination nodules (Shanahan and Hayman, 1990; Schneider et al., 2015).

Histones are protein components of nucleosomes that can undergo post-translational modifications through methylation, acetylation, ubiquitination, sumoylation or phosphorylation of different amino acids present in the amino terminal tail (Venkatesh and Workman, 2015). Such changes reshape the chromatin, promoting the activation or inactivation of genes, or recruiting specific proteins to perform certain functions (Bannister and Kouzarides, 2011). During meiosis I in several organisms, insertions of histone variants in the chromatin are observed; these include the phosphorylated serine 136 histone variant H2AX (γH2AX) (Cabrero et al., 2007) and methylation or acetylation of histone H3 at different amino acids (Hayashi et al., 2005). Such changes may be related to the regulation of several meiotic processes, including recombination, condensation, segregation and transcriptional silencing of homologues (Moens et al., 2007; Naumova et al., 2013; Rahn et al., 2016).

Thus, *Tityus* scorpions may be excellent models for epigenetic studies on achiasmatic meiotic chromosomes. In this study, we performed a detailed analysis of the meiotic cycle of *Tityus silvestris* Pocock, 1988, with the objective of analyzing the synaptic behavior, verifying the occurrence and repair of DSBs and investigating the relationship of these events to epigenetic modifications of bivalents throughout prophase I of this species. In addition, information about *Tityus* chromatin epigenetic dynamics may contribute to understanding the molecular organization of DNA in holocentric systems (Heckmann et al., 2013).

## RESULTS

**Fig. 1.**
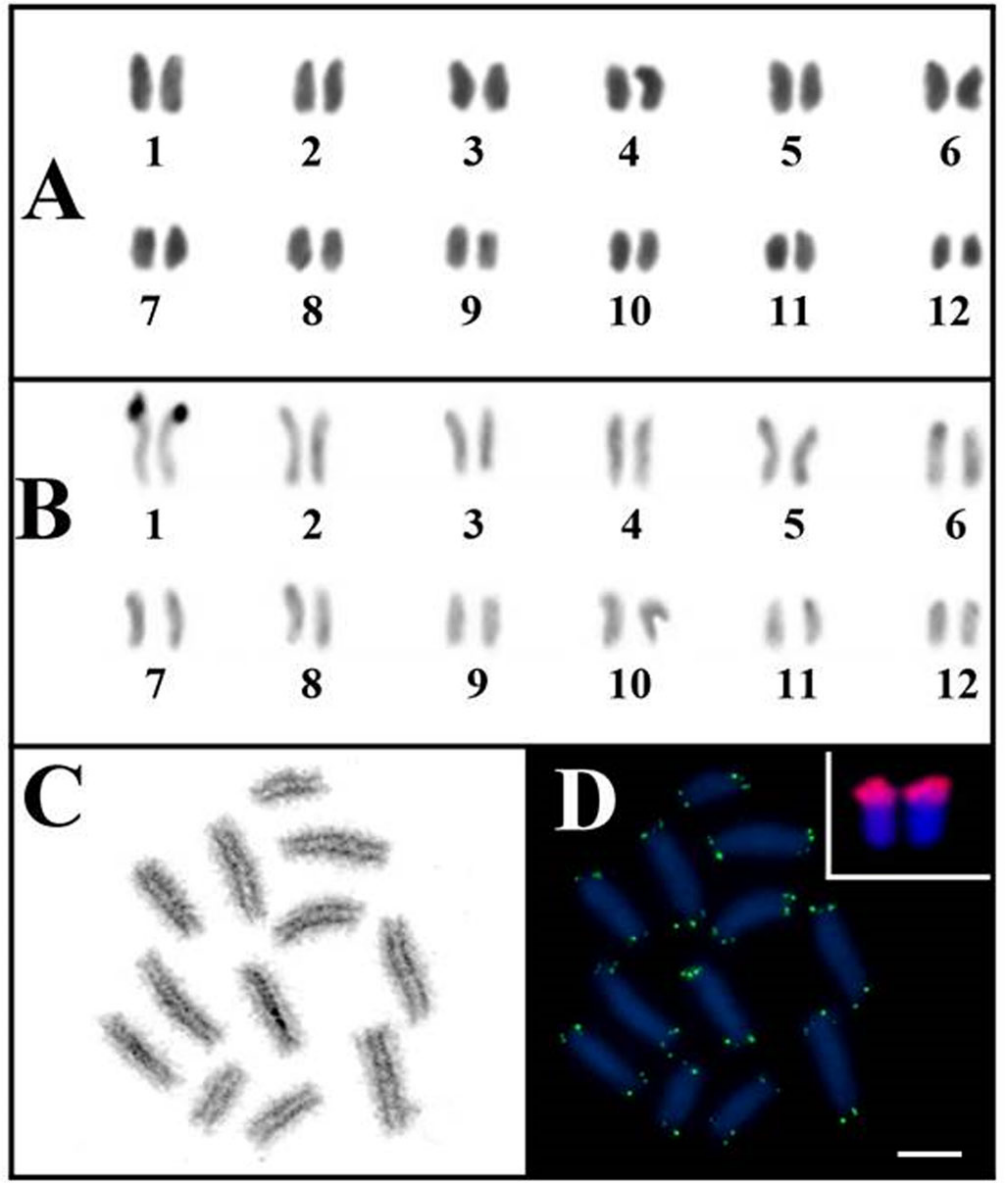
**Cytogenetic characterization of *T. silvestris*.** (A) Karyotype, 2*n*=24. (B) C-banding; note the heterochromatic block in pair 1. (C) Post-pachytene stage meiotic cell demonstrating 12 achiasmatic bivalents. (D) The same post-pachytene stage cell from C submitted to FISH with a telomeric probe; inset shows the rDNA 45S carrier (pair 1). Scale bar: 10 μm.

*Tityus silvestris* has 2*n*=24 holocentric chromosomes, with one large-size pair and 11 medium-size pairs (Fig. 1A). C-banding revealed only one heterochromatic block in the terminal region of pair 1 (Fig. 1B). During meiosis, post-pachytene cells exhibited bivalents, with homologues arranged parallel to each other, without a configuration that evidences the presence of chiasma (Fig. 1C). Fluorescence *in situ* hybridization (FISH) using 45S rDNA showed that this sequence is co-localized with the heterochromatin of pair 1 (Fig. 1D). Telomeric sequences were observed only at chromosomal ends (Fig. 1D).

**Fig. 2.**
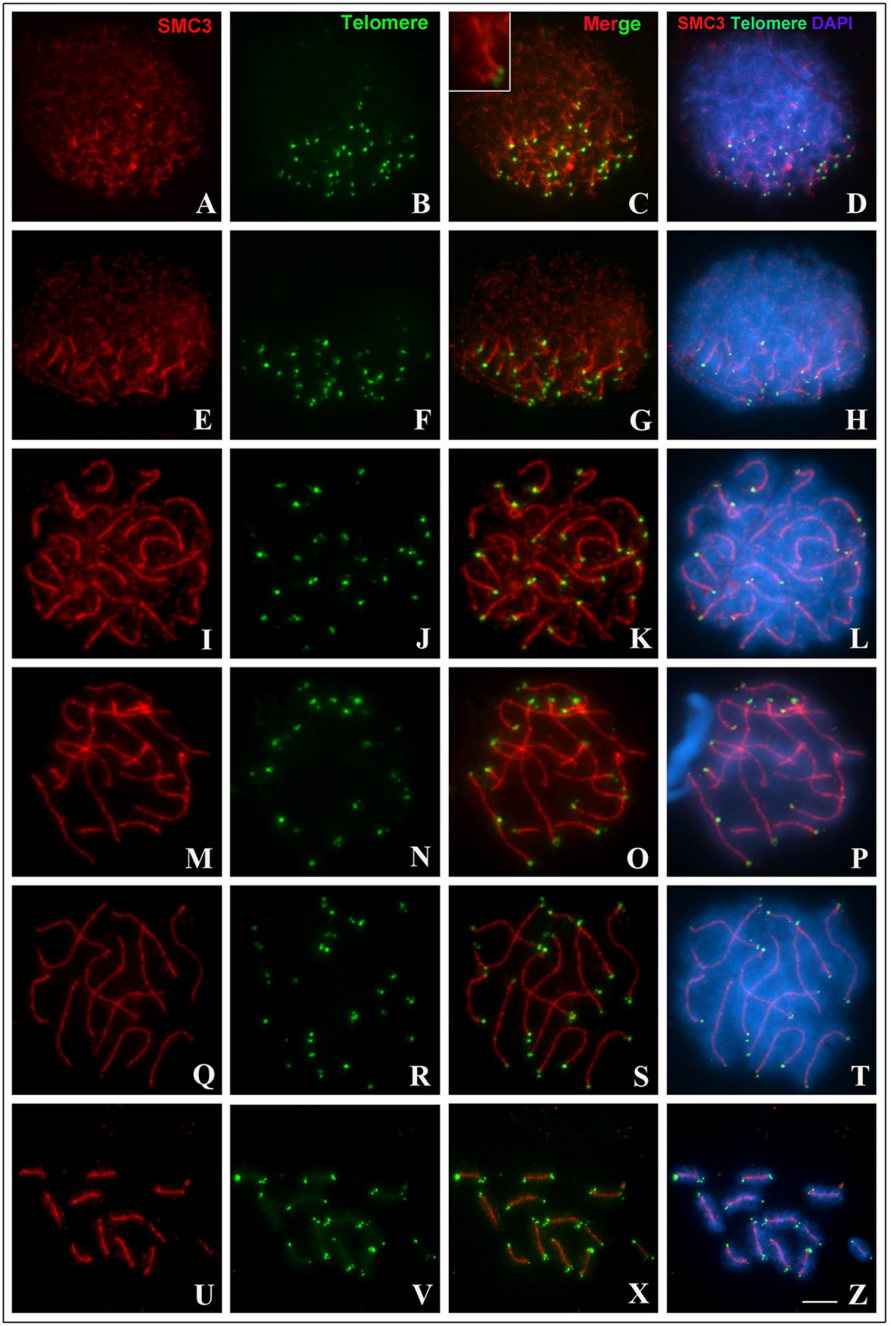
**Synaptic behavior in *T. silvestris* inferred by immunodetection of SMC3 (red) and FISH with telomeric probe (green).** Chromatin was counterstained with DAPI (blue). (A–D) A cell in the late leptotene stage; inset in C demonstrates the pairing of a bivalent. (E–H) A cell in the zygotene stage, with telomeres polarized in a bouquet formation. (I–L) A cell in the early pachytene stage. (M–P) A cell in the intermediate pachytene stage. (Q–T) A cell in the late pachytene stage. (U–Z) A cell in metaphase I; note the maintenance of the SMC3 axial shaft. Scale bar: 10 μm.

Immunodetection of SMC3 (Structural Maintenance of Chromosomes 3) protein and FISH with a telomeric probe showed that, in the leptotene stage, cohesin SMC3 was present in the form of single filaments, dispersed along nuclei, or initiating the bouquet formation (Fig. 2A–D). During the zygotene stage, SMC3 filaments and telomeres were polarized in the bouquet configuration, with synapsis initiation of homologs through terminal regions (Fig. 2E–H). In early pachytene stage, the bouquet configuration became disorganized, exhibiting longer double filaments, owing to advancement of the synapsis, that were continuous with single filaments (Fig. 2I–L). During the intermediate pachytene stage, bivalents were almost totally synapsed, but with interstitial regions that had not yet completed the synapsis process (Fig. 2M–P); the synapsis of bivalents was completed in the late pachytene stage (Fig. 2Q–T). Post-pachytene cells exhibited the presence of 12 completely synapsed bivalents, in which the SMC3 axis remained centrally located along each bivalent to the metaphase I stage (Fig. 2U–Z).

**Fig. 3.**
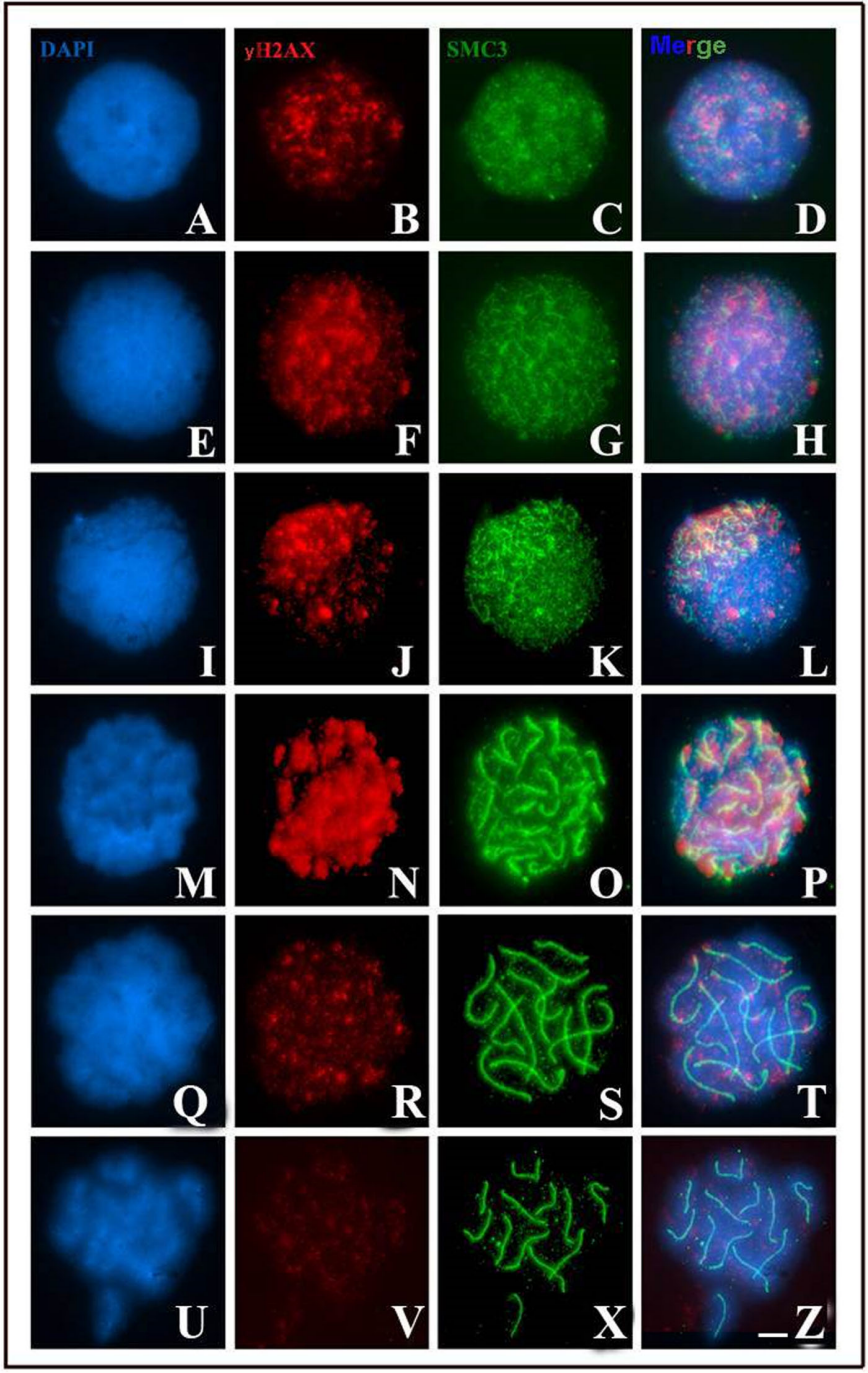
**Signaling of DSBs by γH2AX (red) and SMC3 (green) during prophase I of *T. silvestris*.** Chromatin was counterstained with DAPI (blue). (A–D) A cell in the pre-leptotene stage demonstrating the presence of many γH2AX domains. (E–H) A cell in the leptotene stage. (I–L) A cell in the zygotene stage with γH2AX signals located in the bouquet formation. (M–P) A cell in the early pachytene stage, with γH2AX signals along synapsed axes. (Q–T) A cell in the intermediate pachytene stage with few γH2AX tags. (U–Z) A cell in the late pachytene stage. Scale bar: 10 μm.

Immunodetection of γH2AX demonstrated that the formation of DSBs starts in pre-leptotene cells (Fig. 3A–D). In leptotene cells, γH2AX signals were randomly distributed along the nucleus (Fig. 3E–H). During the zygotene stage, γH2AX signals were co-located with SMC3 axes, polarized according to the bouquet configuration (Fig. 3I–L). In the early pachytene stage, γH2AX signals increased in intensity, expanding along the synapsed SMC3 axes (Fig. 3M–P). In the intermediate pachytene stage, the number of γH2AX signals decreased drastically, with only a few visible domains, most of which were restricted to the bivalent terminal region (Fig. 3Q–T). No γH2AX signals were observed at the late pachytene stage (Fig. 3U–Z).

**Fig. 4.**
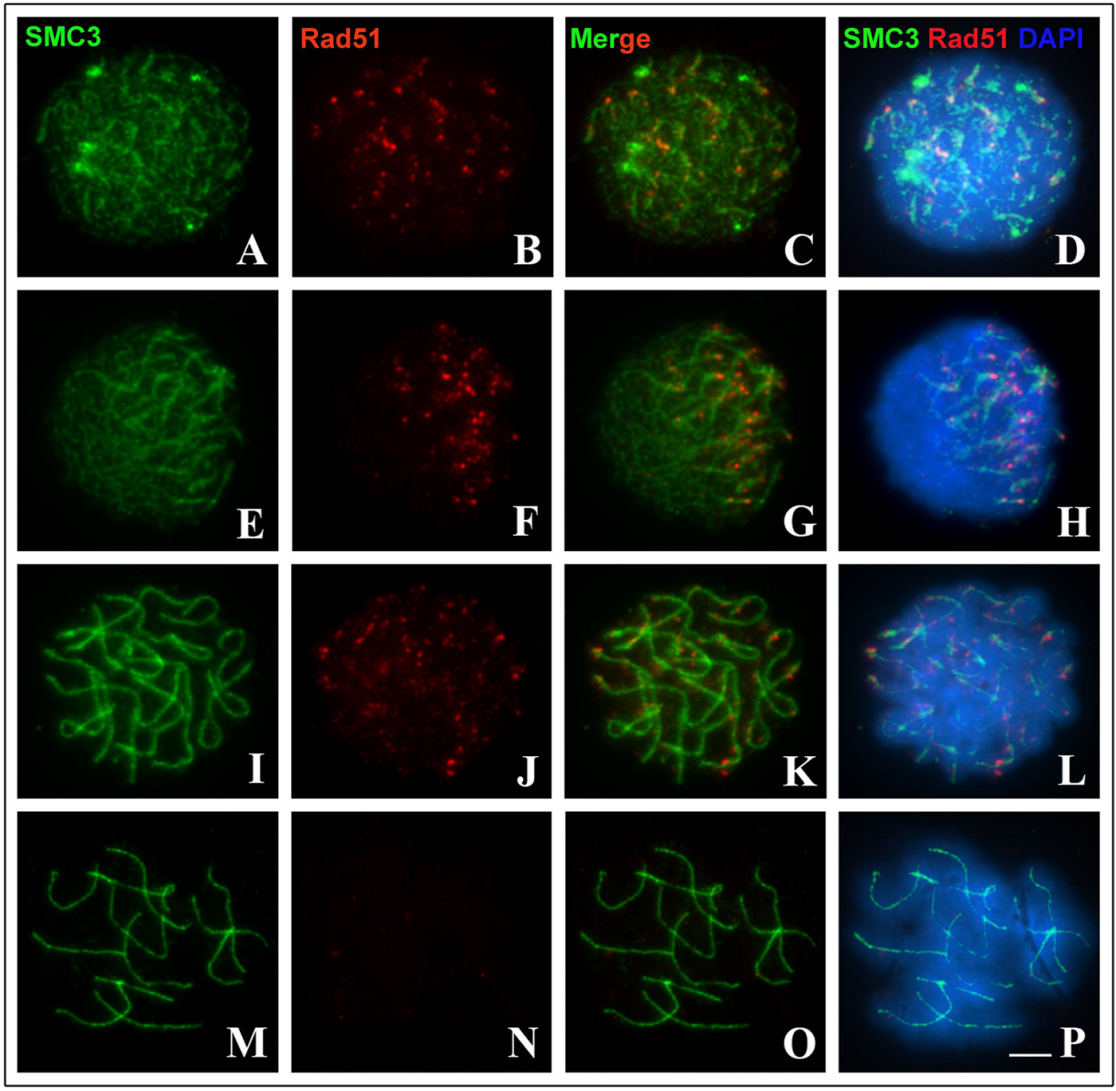
**Repair of DSBs during prophase I of *T. silvestris* inferred by immunodetection of SMC3 (green) and Rad51 (red).** Chromatin was counterstained with DAPI (blue). (A–D) A cell in the leptotene stage demonstrating few foci Rad51. (E–H) A cell in the zygotene stage, with Rad51 on synapsed axes in the bouquet formation. (I–L) A cell in the intermediate pachytene stage with foci Rad51 along the bivalents. (M–P) A cell in the late pachytene stage. Scale bar: 10 μm.

An analysis of the distribution of Rad51 revealed that foci of this recombinase were first detected in the leptotene stage, located on scattered SMC3 axes along the nucleus (Fig. 4A–D). In the zygotene stage, Rad51 was observed only in the synapsed chromosome axes of the bouquet (Fig. 4E–H). During early and intermediate phases of the pachytene stage, Rad5 foci were distributed along the SC, detectable on different regions of the synapsed axes or lateral to them (Fig. 4I–L). No Rad51 signals were observed in the late pachytene stage (Fig. 4M–P).

**Fig. 5.**
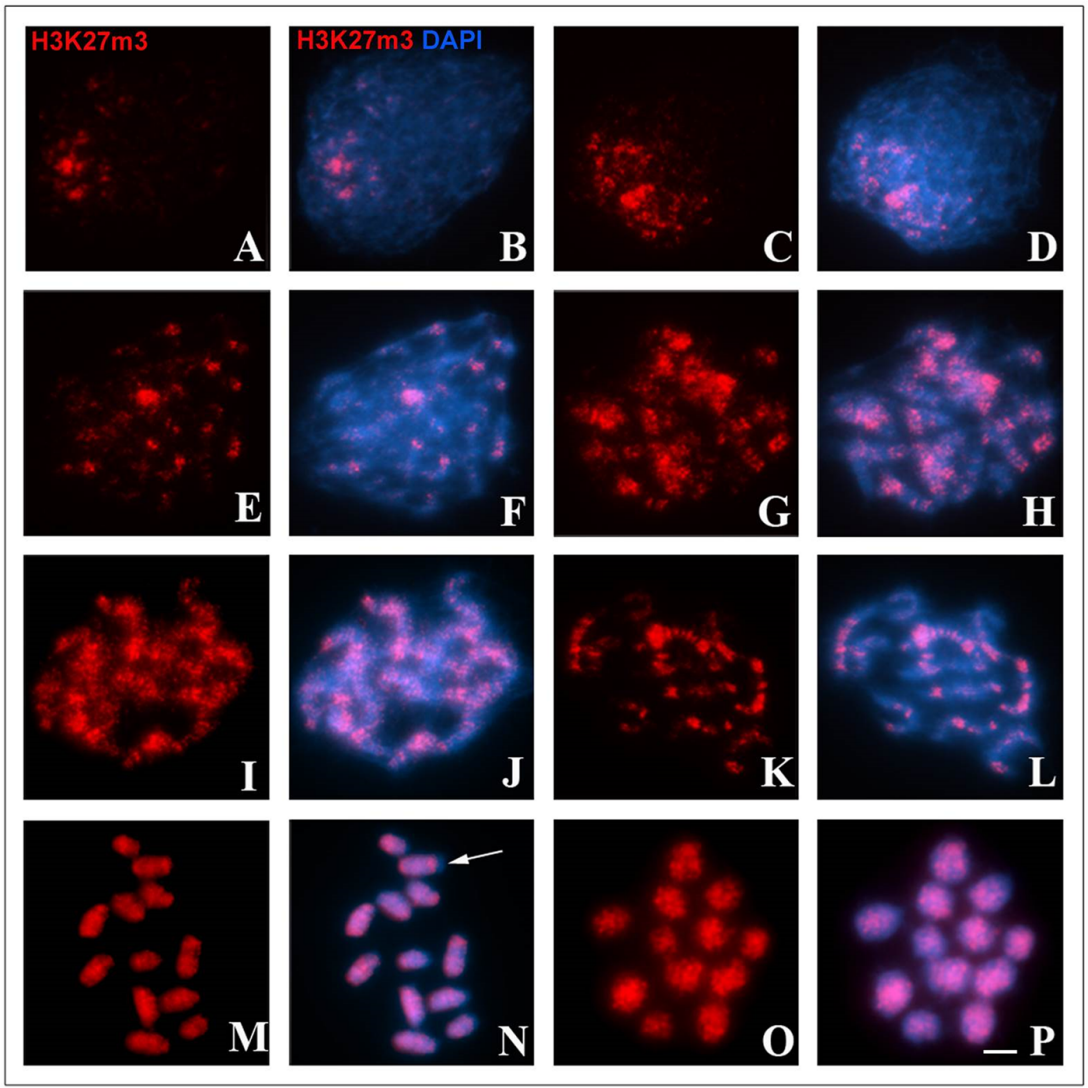
**Dynamics of chromatin rich in H3K27m3 (red) in prophase I of *T. silvestris*.** Chromatin was counterstained with DAPI (blue). (A,B) A cell in the leptotene stage, with few H3K27m3. (C,D) A cell in the early zygotene stage with increase of H3K27m3 in the bouquet formation. (E,F) A cell in the late zygotene stage with decoupling of the bouquet configuration. (G,H) A cell in the early pachytene stage; note the propagation of H3K27m3 along the synapsed axes. (I,J) A cell in intermediate the pachytene stage. (K,L) A cell in the late pachytene stage, with H3K27m3 band-like markings. (M,N) A cell in early metaphase I; the arrow indicates the heterochromatic block with absence of final H3K27m3. (O,P) A cell in metaphase I. Scale bar: 10 μm.

An analysis of the anti-H3K27m3 antibody-labeling pattern demonstrated that the proportion of histones bearing this modification increased in association with advancement of the synapsis. In the leptotene stage, H3K27m3 was located only at one pole of the cellular nucleus (Fig. 5A,B). During the early zygotene stage, the number of H3K27m3 signals increased slightly but were still polarized in the bouquet over thick chromatin, which comprises regions containing initiated synapsis (Fig. 5C,D). In late zygotene stage, the number and intensity of anti-H3K27m3 signals did not change, and were observed mainly on the terminal region of bivalents (Fig. 5E,F). In early pachytene stage, there was a large increase in the amount of H3K27m3 that remained localized only in the synapsed parts of homologs (Fig. 5G,H). In the intermediate pachytene stage, H3K27m3 signals were observed along all bivalents (Fig. 5I,J). In late pachytene cells, on the other hand, the amount of H3K27m3 decreased, and the signals appeared to be compartmentalized, exhibiting a binding-like pattern in some bivalents (Fig. 5K,L). With progression of chromosomal condensation to post-pachytene and metaphase I stages, H3K27m3 signals were observed with a distribution that covered most bivalent extensions, with the exception of the terminal region of some bivalents, including pair 1 carrying the C band (Fig. 5M–P).

**Fig. 6.**
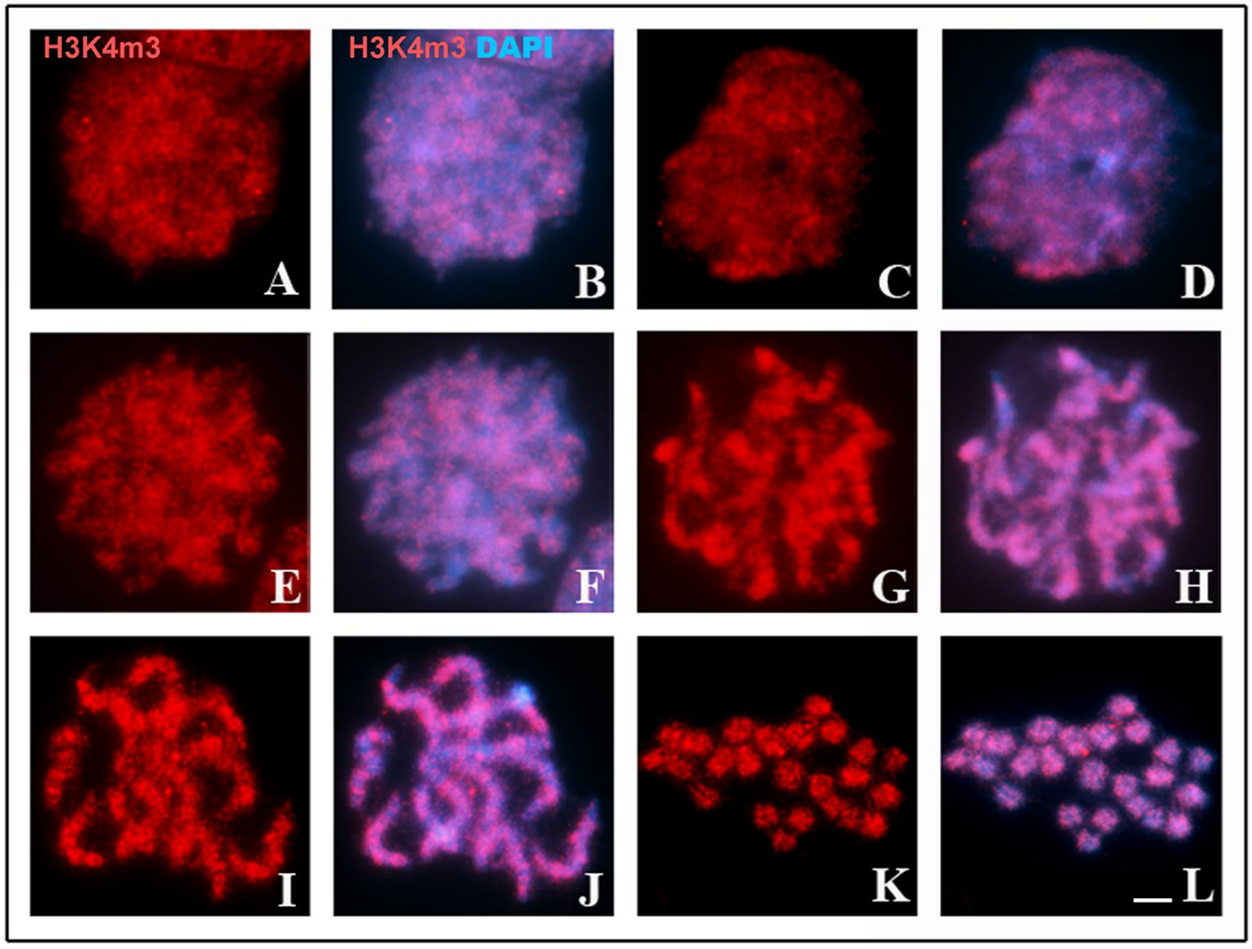
**Distribution of H3K4m3 (red)-rich chromatin during meiosis I of *T. silvestris*.** Chromatin was counterstained with DAPI (blue). (A,B) A cell in the leptotene stage, with the nucleus uniformly labeled with H3K4m3. (C,D) A cell in the early zygotene stage. (E,F) A cell in the late zygotene stage. (G,H) A cell in the early pachytene stage, note the presence of some terminal and interstitial regions without H3K4m3 markings. (I,J) A cell in the late pachytene stage. (K,L) A cell in metaphase II. Scale bar: 10 μm.

Immunodetection of H3K4m3 revealed a uniform distribution of this epigenetic mark along the nucleus of leptotene stage cells (Fig. 6A,B). During the zygotene stage, the pattern was the same as that observed in leptotene cells; however, it was possible to identify some terminal regions that contained few H3K4m3 signals (Fig. 6C–F). In early and late pachytene cells, H3K4m3 signals were detected along almost all extensions of the homologues, where they were present in both synapsed and non-synapsed regions; exceptions included some interstitial and terminal regions that exhibited a low intensity of H3K4m3 signals (Fig. 6G–J). In metaphase II cells, chromosomes exhibited an apparently homogeneous distribution of H3K4m3, and it was not possible to clearly distinguish regions that lacked these modified histones (Fig. 6K,L).

**Fig. 7.**
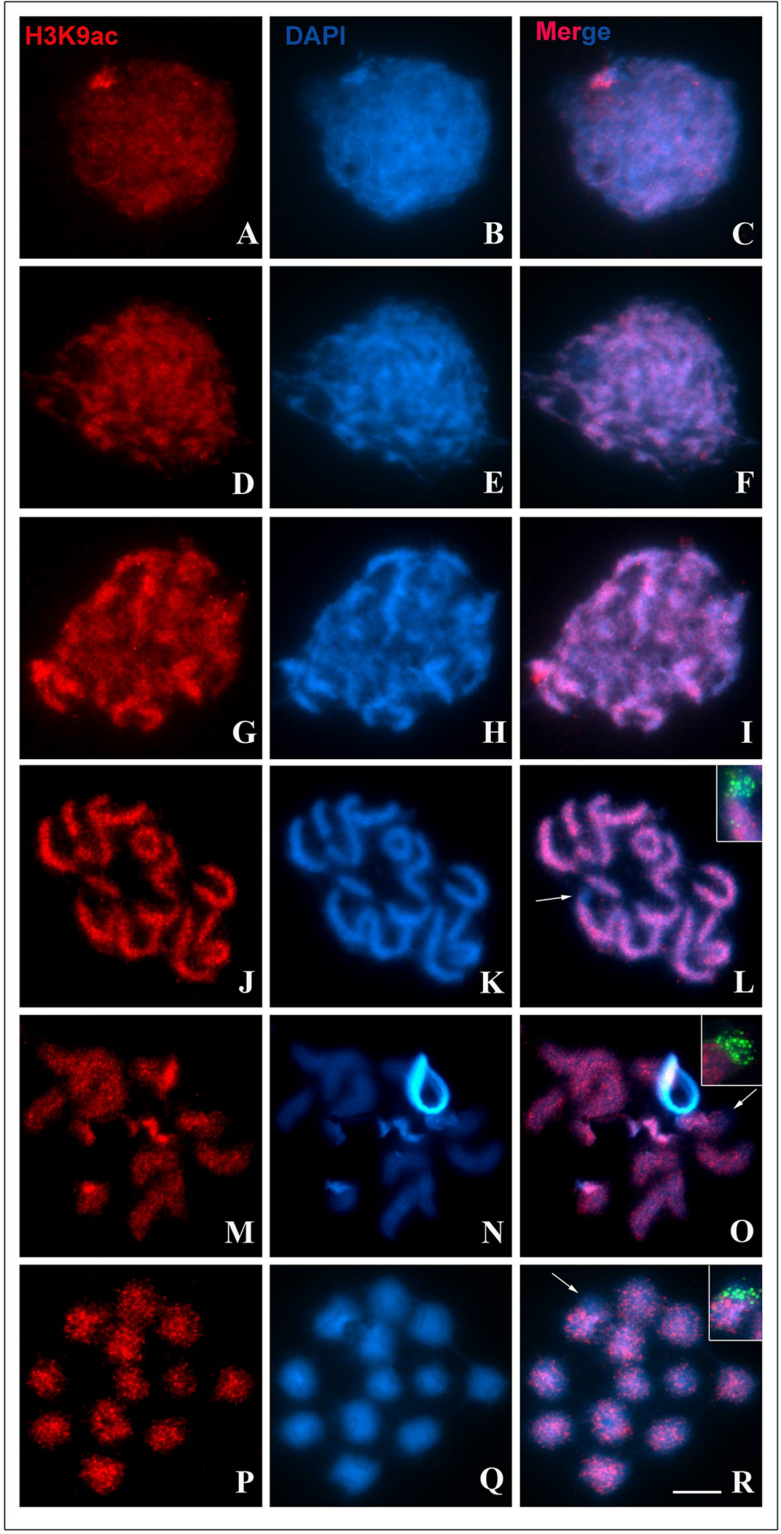
**Distribution of H3K9ac (red)-rich chromatin during meiosis I of *T. silvestris*.** Chromatin was counterstained with DAPI (blue). (A–C) A cell in interphase with heterochromatin rich in H3K9ac. (D–F) A cell in the leptotene stage. (G–I) A cell in the zygotene stage. (J–L) A cell in the intermediary pachytene stage. (M–O) A cell in the post-pachytene stage. (P–R) A cell in metaphase I; arrows (L,O,R) show heterochromatic region with absence of H3K9ac; insets (L,O,R) demonstrate 45S rDNA (green) co-localized with this heterochromatin. Scale bar: 10 μm.

An antibody specific for lysine 9-acetylated histones (H3K9ac) revealed an apparently uniform distribution of this epigenetic mark in interphase chromosomes, with a small accumulation in the heterochromatic region (Fig. 7A–C). In the early stages of prophase I (leptotene and zygotene stages), the distribution of H3K9ac remained uniform throughout the bivalents (Fig. 7D–I). On the other hand, the heterochromatic region of pair 1, identified by the presence of 45S rDNA, did not show H3K9ac signals during pachytene subphases (Fig. 7J–L). This pattern was also observed in post-pachytene and metaphase I cells (Fig. 7M–R). An antibody specific for lysine 27-acetylated histones (H3K27ac) showed no signals for this epigenetic mark in meiotic cells; these signals were only detected in interphase cells, where they were homogeneously distributed (data not shown).

## DISCUSSION

### Synaptic dynamics in *T. silvestris*

In the present study, we demonstrated that synapsis in *T. silvestris* is initiated at terminal regions, arranged in a bouquet configuration, during the leptotene–zygotene transition (Fig. 2). This feature seems to be common for Buthidae members, and suggests that pairing centers are present at chromosome ends in scorpions of this family (Shanahan and Hayman, 1990; Adilardi et al., 2015). This result agrees with findings from other holocentric organisms, such as *Caenorhabditis elegans*, for example, in which each bivalent has a pairing center close to telomeres (Rog and Dernburg, 2013). In Buthidae, terminal regions are composed of several repetitive DNAs present in euchromatin or heterochromatin (Adilardi et al., 2015; Almeida et al., 2017); such sequences may facilitate pairing and synapsis processes, as proposed by Almeida et al. (2017), for histone H3 clusters in *Tityus obscurus*.

The composition and various types of epigenetic modifications of chromatin play important roles in the synapsis (Spangenberg et al., 2010). Immunodetection of H3K27m3 in *T. silvestris* revealed a correlation of this epigenetic mark with synapsis during prophase I (Fig. 5). In mice, meiotic studies have revealed that the enrichment of H3K27m3 occurs in repetitive sequences, which associate with the lateral elements of the SC and act as anchor points for them during the pachytene stage (Hernández-Hernández et al., 2008; Hernández-Hernández et al., 2010). Thus, we believe that, in *T. silvestris*, H3K27m3 signals indicate inactivated sequences associated with the organization of SC, similar to the proposal of Hernández-Hernández et al. (2010) for *Mus musculus*.

In the case of achiasmatic meiosis, pairing and synapsis adaptations accomplish the objective of ensuring correct segregation of homologues in anaphase I. Our results demonstrated that, in *T. silvestris*, SC persist until metaphase I, a finding observed in other species of Buthidae and in some orders of insects (Marec, 1996; Schneider et al., 2009b). In insects, this characteristic is an adaptation related to the achiasmatic mode, and is only possible thanks to the existence of a modified SC (Bogdanov, 2003). Our study demonstrated that the cohesin SMC3 also remains preserved along the SC during metaphase I in *T. silvestris*, diverging from other arthropods in which SMC3 is observed only in the space between chromatids (Calvente et al., 2013) or at centromeres (McKee et al., 2012) during this phase of the meiotic cycle.

### DSB repair and its significance in achiasmatic meiosis in *T. silvestris*

Achiasmy is a phenomenon that has arisen in several independent eukaryotic lines, usually associated with heterogametic sex (Loidl, 2016). Although meiosis is achiasmatic in *T. silvestris*, DSBs form and are repaired, as evidenced by the detection of γH2AX and Rad51 proteins (Figs 3 and 4). Similar results have been found only in *Rhynchospora tenuis* and *Bombyx mori* females, in which initial recombination nodules are observed during achiasmatic meiosis (Holm and Rasmussen, 1980; Cabral et al., 2014). In the specific case of *T. silvestris*, we suggest that DSBs are not repaired in the form of crossing-over. The absence of late recombination nodes in ultrastructural analyses of the SC of four Buthidae species supports our hypothesis that DSBs are processed by non-crossing-over mechanisms in *T. silvestris* (Schneider et al., 2015). The absence of chiasma in *T. silvestris* is considered an advantageous characteristic that was maintained during the chromosomal evolution of Buthidae, possibly because it allows normal segregation of the chromosomes involved in multivalent meiosis (Mattos et al., 2013; Adilardi et al., 2016; Almeida et al., 2017).

Considering this information, what purpose might DSBs serve during prophase I in *T. silvestris*? According to Zickler (2006), homologous pairing occurs in three distinct and consecutive steps in most eukaryotes: homologous recognition, juxtaposition of chromosomal axes mediated by DSBs and SC formation. Considering the results of the present study, we believe that the maintenance of DSBs in *T. silvestris* is attributable to the role played by these events in the synaptic mechanism. In this scorpion, this relationship between DSB dependence and synapsis is corroborated by the chronology of these mechanisms during prophase I. This is indicated by our immunocytochemical experiments (Figs 2, 3 and 4), which demonstrated that the synapsis starts in the zygotene stage, after the appearance of DSBs, which occurs during the leptotene stage, following the general model of eukaryotes (Zickler, 2006). Accordingly, the maintenance of DSB formation and repair mechanisms may be essential for the synaptic process in this species.

### Regulation of transcription in heterochromatin during prophase I in *T. silvestris*

Recent studies in mice and yeast have provided evidence for the enrichment of both H3K4m3 and H3K9ac epigenetic marks, typical of transcriptionally active chromatin, at DSBs sites (Lichten and De Massy, 2011; Yamada et al., 2013). In this case, it is thought that these histone modifications serve to decompact the chromatin, making it accessible to Spo11 and other components of the recombination machinery, or to recruit chromatin remodelers (Borde et al., 2009; Getun et al., 2017). In the present study, we found that H3K4m3 and H3K9ac marks were equally distributed throughout the initial phases of prophase I in *T. silvestris* (Figs 6 and 7), a pattern that exhibits no relationship between them and the formation of DSBs. In *Saccharomyces cerevisiae*, the absence of H3K4m3 modifications does not prevent formation of DSBs (Borde et al., 2009). The observed patterns of the distribution of H3K4m3 and H3K9ac marks in *T. silvestris* (Figs 6 and 7) may represent the axial axis loops of chromosomes that are actively engaged in the process of transcription (Prakash et al., 2015), a suggestion that aligns well with results of recent studies on transcriptional activity during Arthropoda meiosis. In *Drosophila*, for example, transcription is maintained constant throughout meiosis I (Hennig and Weyrich, 2013). In Hemiptera, Viera et al. (2017) reported that transcription is reactivated during the zygotene stage and proceeds until the end of prophase I in autosomes.

In *T. silvestris*, H3K27m3 modifications are reported to be absent in the heterochromatin region located in pair 1 during metaphase I (Fig. 5). This result can be explained by the presence of other histone marks in the positive C-block of this species. In eukaryotes, several studies have shown that the formation of constitutive heterochromatin (pericentromeric or terminal) is related to the tri-methylation of histone H3 in lysine 9 (H3K9m3) (Nishibuchi and Déjardin, 2017). This epigenetic modification is recognized by the protein HP1 (Heterochromatin Protein 1), which performs the process of heterochromatinization (Saksouk et al., 2015). Meiotic analyses in grasshoppers (Viera et al., 2009), crustaceans (Gómez et al., 2016) and Hemiptera (Viera et al., 2009) have revealed an H3K9m3 distribution pattern in which this modification is co-localized with constitutive heterochromatin. Thus, we believe that the C-positive heterochromatin in *T. silvestris* is enriched for H3K9m3.

In our study, we observed that H3K9ac was absent in the constitutive heterochromatin of pair 1 in *T. silvestris* after the pachytene stage (Fig. 7). These results suggest that this epigenetic mark participates in the regulation of 45S rDNA and is active during leptotene and zygotene stages, before being silenced later in the pachytene stage. These data are in agreement with literature reports on the transcriptional activity of this sequence during prophase I (see Hernandez-Verdun, 2011) and demonstrate that H3K9ac is a good marker for 45S rDNA expression studies in Arthropoda.

Our study demonstrated that the synapsis in *T. silvestris* begins at chromosomal ends and its progression is directly related to the trimethylation of histone H3 lysine 27. In *T. silvestris*, DSB formation and repair are not related to sites rich in H3K4m3 or H3K9ac, and their simultaneous occurrence with achiasmatic meiosis demonstrates that programmed DNA breaks are not processed through homologous recombination, but may be important for the synaptic mechanism of this species. The presence of the epigenetic marks, H3K4m3 and H3K9ac, demonstrates constitutive gene activity throughout prophase I; in turn, the absence of H3K9ac in constitutive heterochromatin carrying the 45S rDNA from the pachytene stage indicates silencing of this gene in this phase of meiosis I.

## MATERIALS AND METHODS

Six individuals (four male and two female) of *T. silvestris*, collected in the municipality of Belém, Pará, Brazil (1°24′16.29″S/48°27′12.29″W), were used as samples in this study. For karyotype analysis, testes and ovaries were processed according to the protocol described by Schneider et al. (2009a,b). Chromosomal preparations were stained with 5% Giemsa. C-banding was performed according to Sumner (1972).

Meiotic preparations for obtaining SC were generated according to the method of Viera et al. (2009), with some modifications. Testes were removed and held in Hanks's solution for 10 min, and then hypotonized in 0.075 M KCl for 30 min. Thereafter, gonads were transferred to a 100-mM sucrose solution (pH 8.5), in which they were disrupted with the aid of needles. Approximately 40 μl of the generated cell suspension was spread on slides, precoated with 2% paraformaldehyde (pH 8.5) containing 0.15% Triton-X. The slides were dried in a humid chamber at room temperature for 2 h, then washed in 0.08% Photo-Flo (Kodak), and stored at −80°C.

For immunodetection of meiotic proteins, slides were washed three times in phosphate-buffered saline (PBS), followed by blocking with a 5% bovine serum albumin solution at room temperature for 30 min. Primary antibodies used and their respective dilutions in PBS were as follows: rabbit anti-SMC3 (Abcam, ab9263) at 1:200; rabbit anti-γH2AX (Abcam, ab2893) at 1:50; rabbit anti-Rad51 (Santa Cruz Biotechnology, H92 sc-8349) at 1:50; rabbit anti-H3K27m3 (Cell Signaling, 9733S) at 1:50; rabbit anti-H3K27ac (Millipore, 07-360) at 1:50; rabbit anti-H3K4m3 (Millipore, 07-473) at 1:50; and rabbit anti-H3K9ac (Millipore, 07-352) at 1:50. Slides were incubated with antibodies for 2 h at 37°C in a humidified chamber. After washing in PBS, slides were incubated for 2 h at 37°C with the appropriate secondary antibodies, diluted in 1:100 PBS-Tween. Chromatin was counterstained by incubating with Vectashield Antifade Mounting Medium containing DAPI (4′,6-diamidino-2-phenylindole).

FISH technique and the production of telomeric and 45S rDNA probes were performed according to Almeida et al. (2017), with few modifications. Slides used for immunodetection were washed three times in 4×SSC-Tween, and then dehydrated using an ethanol series (70%, 90% and 100%). The probes and chromosomal DNA were denatured at 100°C and 70°C, respectively. Hybridization was performed by incubating overnight at 37°C. Probe was detected by incubating with a fluorescein isothiocyanate-conjugated anti-digoxigenin antibody at 37°C for 1 h. Chromatin was counterstained by incubating with Vectashield Antifade Mounting Medium containing DAPI.

### Ethical approval

All applicable international, national and/or institutional guidelines for the care and use of animals were followed. J.C.P. has a permanent field permit, number 13248, from Instituto Chico Mendes de Conservação da Biodiversidade. The Cytogenetics Laboratory from Federal University of Pará has permit number 19/2003 from the Ministry of Environment for sample transport and permit 52/2003 for using the samples for research. The Ethics Committee (Comitê de Ética Animal da Universidade Federal do Pará) approved this research (Permit 68/2015).
